# Liquid Biopsy of Extracellular Vesicle-Derived miR-193a-5p in Colorectal Cancer and Discovery of Its Tumor-Suppressor Functions

**DOI:** 10.3389/fonc.2020.01372

**Published:** 2020-08-18

**Authors:** Rui Wei, Lei Chen, Da Qin, Qingdong Guo, Shengtao Zhu, Peng Li, Li Min, Shutian Zhang

**Affiliations:** Beijing Key Laboratory for Precancerous Lesion of Digestive Disease, Department of Gastroenterology, National Clinical Research Center for Digestive Disease, Beijing Digestive Disease Center, Beijing Friendship Hospital, Capital Medical University, Beijing, China

**Keywords:** extracellular vesicle, miR-193a-5p, plasma, colorectal cancer, metastasis, biomarker

## Abstract

Previously, abnormal extracellular vesicle (EV) sorting of miR-193a was identified in colorectal cancer (CRC) progression. Although a reduced level of miR-193a-5p in plasma/serum has been reported in many different types of cancer, the EV-derived miR-193a-5p level in CRC and its potential application as a minimally invasive biomarker are still unknown. Here, we evaluated the circulating EV-derived miR-193a-5p expression levels in a cohort of 101 participants by real-time quantitative polymerase chain reaction (RT-qPCR). We found that plasma EV-miR-193a-5p decreased significantly in CRC patients as compared with precancerous colorectal adenoma (CA) and non-cancerous control (NC) individuals. The circulating EV-miR-193a-5p showed an area under the receiver operating characteristic curve (AUC) of 0.740 in distinguishing CRC from CA and an AUC of 0.759 in distinguishing CRC from NC. Furthermore, the suppression on CRC cells of miR-193a-5p was verified by transwell, MTS (3-(4,5-dimethylthiazol-2-yl)-5-(3-carboxymethoxyphenyl)-2-(4-sulfophenyl)-2*H*-tetrazolium), EdU, RT-qPCR, and western blotting. Bioinformatic analysis predicted 32 genes, which were the most likely miR-193a-5p targeted and mainly focused on tumor progression. Among them, we revealed that miR-193a-5p could inhibit CRC migration and invasion via targeting tumor-associated genes like CUT-like homeobox 1 (CUX1) and intersectin 1 (ITSN1). In conclusion, miR-193a-5p could suppress CRC development, and decreased plasma EV-miR-193a-5p could be a promising biomarker for human CRC detection.

## Introduction

Colorectal cancer (CRC) with distant metastasis and local invasion is associated with a poor prognosis (1). Combined treatment that includes surgery, chemotherapy, and radiation may improve clinical outcomes but is not curative (2). It is generally accepted that the 5-year survival rate of CRC is very closely associated with the TNM stage at detection (3). Early detection through frequent examinations, including endoscopic and radiologic examinations, is the key to improve prognosis. Easy and minimally invasive diagnostic methods, such as blood and urine CRC screening, are emerging but not satisfactory (4). Currently, serological tumor biomarkers such as carcinoembryonic antigen (CEA) and carbohydrate antigen 19-9 (CA19-9) have been clinically used for the detection of many cancers including CRC (5, 6), yet with low specificity (7–9). Thus, the development of new minimally invasive strategies is urgently needed to identify CRC patients at an early stage.

MicroRNAs (miRNAs) are small non-coding RNAs (ncRNAs) that regulate post-translation of protein-coding messenger RNAs (mRNAs) (10, 11). Several studies have revealed that miRNAs play an important role in carcinogenesis through oncogenic or tumor-suppressor functions, depending on the downstream targets they regulated. The identification of circulating miRNAs in the serum or plasma indicated that miRNAs may be potentially useful as clinical diagnostic or prognostic tools (12, 13).

Extracellular vesicles (EVs) are 30- to 100-nm nanoscale vesicles secreted by specific cell types, including T cells, platelets, and cancer cells, and concentrated in bodily fluids (14, 15). With an intact phospholipid bilayer structure, EVs have the capacity of protecting their inner miRNAs from endogenous RNase activity. Thus, they can maintain the integrity of miRNAs in circulation and function as efficient minimally invasive biomarkers for different cancers (12, 16–18). Recently, several studies have reported the predictive potential of EV-miRNAs from plasma/serum for early detection in different cancers (19–21). For instance, EV-miR-1260b in plasma was upregulated in lung adenocarcinoma and promoted cancer cell invasion by regulating Wnt/β-catenin signaling pathway (22). Low EV-miR-548c-5p in serum was associated with poor prognosis in CRC (23).

MiRNA-193a-5p (miR-193a-5p), located on 17q11.2, functioned as a tumor suppressor in CRC and other tumors (24–26). Currently, several studies identified a decreased level of plasma/serum miR-193a-5p in CRC with lymph node metastasis, hepatocellular carcinoma, and ovarian cancer (27–29). However, no related research of circulating EV-derived miR-193a-5p has been reported. In this study, we identified the distinct gene expression level of EV-miR-193a-5p in plasma isolated from 101 CRC patients and healthy controls. Our results revealed that the decrease of miR-193a-5p in the plasma EV is a novel blood-based cancer biomarker. We also investigated the relationship between miR-193a-5p level and multiple clinicopathological characteristics, and we confirmed its diagnostic potential in early-stage CRC and precancerous colorectal adenoma (CA) patients. Furthermore, we revealed that miR-193a-5p suppressed CUT-like homeobox 1 (CUX1) and intersectin 1 (ITSN1) expression at posttranscriptional level, thereby regulating epithelial–mesenchymal translation (EMT) signaling pathway and finally inhibiting CRC cell migration and invasion. Here, we identified plasma EV-miR-193a-5p as a potential prognostic marker for CRC patients.

## Materials and Methods

### Study Design

All blood specimens were acquired from patients before endoscopic submucosal dissection or surgical removal of colorectal tumor at Beijing Friendship Hospital, Capital Medical University, from January 2017 to June 2018. With pathological confirmation, 37 patients were diagnosed as CRC, and 22 patients were confirmed as CA. Additionally, 42 non-cancerous control (NC) individuals were recruited from outpatients of the Department of Gastroenterology, Beijing Friendship Hospital. The clinical and pathological data, including diagnosis, age, gender, tumor size, tumor stage, location, depth of infiltration, lymph node metastasis, and morphological classification (such as Yamada and Paris subtypes), were all extracted from clinical record, endoscopic report, and pathological reports. CRC patients were staged by the 8th TNM classification of American Joint Committee on Cancer (AJCC)/Union for International Cancer Control (UICC) staging system. Peripheral blood samples were centrifuged at 3,000 × g for 15 min at 4°C for the preparation of plasma samples. All plasma samples were stored at −80°C for subsequent experiments. Detailed clinicopathological data are summarized in Table 1. All participants in this study had signed the informed consent before the study. This study was approved by the ethics committee of Beijing Friendship Hospital.

**Table 1:** Clinical characteristics of all participants.

**Clinical characteristics**	**CRC** **(*n* = 37)**	**CA** **(*n* = 22)**	**NC** **(*n* = 42)**
Age, *n* (%)			
≤55 years	6 (16.2)	8 (36.4)	22 (52.4)
>55 years	31 (83.8)	14 (63.6)	20 (47.6)
Gender, *n* (%)			
Male	28 (75.7)	19 (86.4)	32 (76.2)
Female	9 (24.3)	3 (13.6)	10 (23.8)
Tumor size, *n* (%)			
≤2 cm	11 (29.7)	13 (59.1)	/
>2 cm	26 (70.3)	9 (40.9)	/
Invasive depth, *n* (%)			
Mucosa	14 (37.8)	22 (100.0)	/
Submucosa	23 (62.2)	0 (0.0)	/
Number of lesions, *n* (%)			
Single site	2 (5.4)	1 (4.5)	/
Multiple site	13 (35.1)	21 (95.5)	/
Not clear	22 (59.5)	0 (0.0)	/
Clinical stage, *n* (%)			
I	18 (48.6)	/	/
II	9 (24.3)	/	/
III	8 (21.7)	/	/
Not clear	2 (5.4)	/	/
Lymph node metastasis, *n* (%)			
N	28 (75.7)	22 (100.0)	/
Y	9 (24.3)	0 (0.0)	/
Tumor location, *n* (%)			
Right colon	8 (21.6)	5 (22.7)	/
Left colon	17 (45.9)	9 (40.9)	/
Rectum	11 (29.8)	5 (22.7)	/
Not clear	1 (2.7)	3 (13.7)	/
Yamada subtype, *n* (%)			
I	5 (13.5)	7 (31.8)	/
II	5 (13.5)	7 (31.8)	/
III	3 (8.1)	4 (18.2)	/
IV	2 (5.4)	4 (18.2)	/
Not clear	22 (59.5)	0 (0.0)	/
Adenoma differentiation status, *n* (%)			
CA-L	/	4 (18.2)	/
CA-H	/	15 (68.2)	/
SSA	/	3 (13.6)	/
Clinical diagnosis, *n* (%)			
Gastritis	/	/	21 (53.3)
Cholelithiasis	/	/	5 (11.1)
PHT (portal hypertension)	/	/	4 (8.9)
Polyps	/	/	5 (11.1)
Others	/	/	7 (15.6)

*CRC, colorectal cancer; CA, colorectal adenoma; SSA, sessile serrated adenoma*.

### Cell Lines

One normal colon epithelial cell (CCC-HIE-2) and two colon cancer cell lines (HCT-8 and SW480) from the American Type Culture Collection (ATCC) were cultured in 10% fetal bovine serum (FBS)-containing Dulbecco's modified Eagle's medium (DEME) media (Gibco, USA) under 5% CO_2_ at 37°C. Cell lines used in our experiments were performed in less than five passages.

### Isolation of Extracellular Vesicles From Human Plasma

EVs from plasma samples were isolated following standard ultracentrifugation procedure reported by previous studies (30, 31). In brief, plasma samples were initially centrifuged at 3,000 × g for 15 min to remove cell debris and 13,000 ×g for 30 min to deplete large particles. We then purified the obtained supernatants by ultracentrifugation at 150,000 ×g for 4 h at 4°C and washed the pellets with phosphate-buffered saline (PBS).

### Identification of Plasma Extracellular Vesicles

We used transmission electron microscopy (TEM) and nanoparticle tracking analysis (NTA) along with western blotting for EV identification. TEM was performed according to standard procedure. The copper grid incubated with EV suspension and furtherly processed was observed using an electron microscope (JEOL-JEM1400, Japan). In order to identify the exact size and movement of the isolated particles, the EV suspension was examined by ZetaView PMX 110 (Particle Metrix, Germany) and video recorded for further movement analysis using the NTA software (ZetaView 8.02.28). Special markers for extracellular vesicles have been previously reported. Here, we used a combination of two positive markers (CD9 and CD63) and one negative marker (GM130) to characterize the EVs we extracted. The protein bands were detected using an enhanced chemiluminescence system (Bio-Rad, USA).

### Extraction of Total RNAs From Plasma Extracellular Vesicles

Total EV-RNAs were extracted according to the protocol of miRNeasy® Mini kit (Qiagen, cat. No. 217004, Germany) and stored at −80°C for subsequent experiments. Briefly, plasma EVs were diluted with 1 ml of lysis reagent. Total RNAs including miRNAs were purified from the lysed products on the basis of the manufacturer's protocol and used as a template for RT-qPCR. The quality of total RNAs was assayed by 1.5% agarose gel electrophoresis and RNA Nano 6000 Assay Kit of the Agilent Bioanalyzer 2100 System (Agilent Technologies, USA).

### RT-qPCR for Plasma EV-miR-193a-5p

Expression of plasma EV-miR-193a-5p was determined by RT-qPCR. TaqMan™ advanced miRNA assays were performed for miRNA quantification using Life TaqMan Advanced miRNA cDNA Synthesis Kit (Life Tech, CA, cat. A28007) and Life TaqMan Fast Advanced Master Mix (Life Tech, CA, cat. 4444557) according to the manufacturer's protocol. *Caenorhabditis elegans* cel-39-3p was added as an external control for RT-qPCR analysis. A specific miR-193a-5p probe was applied for PCR (477954 for miR-193a-5p, Life Technologies, USA).

### Transfection Assay

HCT-8 and SW480 cells were seeded into 6-well plates till 75% confluence. MiR-193a-5p mimics and non-silencing negative control (miR-NC) were synthesized in Suzhou GenePharma Co., Ltd (China) and transfected into two CRC lines by using Lipofectamine 3000. The transfection efficacy was confirmed by RT-qPCR. Target sequences of miR-193a-5p mimics and miR-NC utilized in the study are listed as follows:

Mimics, forward: UGGGUCUUUGCGGGCGAGAUGA;

      reverse: AUCUCGCCCGCAAAGACCCAUU.

miR-NC, forward: UUCUCCGAACGUGUCACGUTT;

      reverse: ACGUGACACGUUCGGAGAATT.

### Cell Migration and Invasion Assays

Migration assay was performed using transwell chamber without Matrigel (8 mm, Corning Costar, USA). Invasion assay was conducted using chamber with Matrigel; 750 μl of DMEM with 10% FBS was in each well of 24-well plate. For HCT-8 cells, 3 × 10^5^ cells were resuspended in 500 μl of FBS-free medium and seeded into the upper chamber slowly, with 2 × 10^5^ for SW480 cell line. The incubation time is 36 h for both cell lines. After being fixed with methanol, cells were stained with methylrosanilinium chloride solution and then photographed with microscope.

### Wound Healing Assay

After transfection for 48 h in 6-well plate, cells were wounded using a sterile pipette tip and washed with PBS to clear out cellular debris. Then cells were continued to culture with DMEM without FBS. Pictures at the time point of 0, 12, 24, 36, and 48 h were taken for assessing the migration status.

### Cell Viability Assay

To explore the function role of miR-193a-5p on the proliferation of cells, one-step MTS (3-(4,5-dimethylthiazol-2-yl)-5-(3-carboxymethoxyphenyl)-2-(4-sulfophenyl)-2*H*-tetrazolium) assays were performed. A total of 3,000 cells/well in 100 μl of medium were seeded in a 96-well plate after transfection and added with 20 μl of MTS reagent/well at the time points of 0, 24, 48, and 72 h. After being incubated at 37°C for 2 h, enzyme-labeled meter (SpectraMax M3, Molecular Devices, USA) was applied to access the cell viability. Three independent experiments were performed in all assays.

### EdU Incorporation Assay

For EdU assays (RiboBio, China), cells were attached to the 24-well plate for 24 h and then cultured with 10 nM of EdU solution at 37°C for 2 h and followed by fixation in 4% formaldehyde for 30 min. Then, the cells were treated with an Apollo cocktail for 50 min and subsequently treated with Hoechst 33342 for 40 min for nuclear staining. Finally, cell proliferation was detected under an inverted fluorescence microscope (Carl Zeiss Axio Observer Z1, Germany). Three independent experiments were performed in all assays.

### Western Blotting Analysis

Radioimmunoprecipitation assay (RIPA) lysis buffer (KeyGen Biotech, China) was used for protein extraction. After protein quantification by the bicinchoninic acid (BCA) protein assay kit (Thermo Fisher, USA), a total of 30 μg denatured proteins per line underwent electrophoresis. Then the proteins were transferred to polyvinylidene fluoride (PVDF) membranes and blocked by 5% milk (non-fat milk in TBST). Membranes were incubated in primary antibodies against N-cadherin, Vimentin, MMP2, CUX1, ITSN1, and GAPDH at 4°C overnight. Antibodies used in the study are listed in Table 2. The following day, after being washed with TBST for three times, membranes were incubated with secondary antibodies for 1 h at room temperature. After washing with TBST for six times, the detection of protein bands was performed with the enhanced chemiluminescence system (Bio-Rad, USA).

**Table 2:** Information of primary antibodies in western blotting (WB).

**Primary antibody**	**Company**	**Cat**.	**Dilution factor**
CD9	Abcam	ab92726	WB 1:2,000
CD63	Abcam	ab217345	WB 1:1,000
GM130	Proteintech	11308-1-AP	WB 1:2,000
N-cadherin	Proteintech	22018-1-AP	WB 1:2,000
Vimentin	Proteintech	10366-1-AP	WB 1:2,000
MMP2	Proteintech	10373-2-AP	WB 1:2,000
CUX1	Proteintech	11733-1-AP	WB 1:2,000
ITSN1	Proteintech	21862-1-AP	WB 1:2,000
GAPDH	Abbkine	A01020	WB 1:5,000

### RNA Extraction and Real-Time Quantitative PCR of Colorectal Cancer Cell Lines

Total RNA was extracted using TRIzol (Invitrogen, Germany) from two cell lines. RT-qPCR was performed using SYBR green mix (Invitrogen, Germany) and run in the 7500 Real-Time PCR Systems (Applied Biosystems, USA) with cycling parameters listed as follows: 94°C for 2 min followed by 40 cycles of 94°C for 15 s, 56°C at 20 s, and 72°C at 30 s and then followed by 72°C for 2 min. With a melting curve analysis, 2^−ΔΔCT^ was used to calculate the relative gene expression of RT-qPCR. Primers detected in the study were listed as follows: miR-193a-5p forward: CTGGGTCTTTGCGGGCGAG; reverse: GAATACCTCGGACCCTGC. miR-193a-5p RT primer: CTCAACTGGTGTCGTGGAGTCGGCAATTCAGTTGAGCTCATCTCG. U6 forward: CTCGCTTCGGCAGCACA; reverse: AACGCTTCACGAATTTGCGT. U6 RT-Primer: AACGCTTCACGAATTTGCGT. GAPDH forward: GGAGCGAGATCCCTCCAAAAT; reverse: GGCTGTTGTCATACTTCTCATGG. CUX1 forward: GAAGAACCAAGCCGAAACCAT; reverse: AGGCTCTGAACCTTATGCTCA. ITSN1 forward: ATTTATCCTGGCAATGCACCTC; reverse: TCCCGCTTCTTATCTTCAAACG.

### Dual-Luciferase Reporter Assay

Cells were seeded into the 24-well plate till 75% confluence. Lipofectamine 3000 (Invitrogen, Germany) was utilized for co-transfection of the CRC cells with miR-193a-5p mimics (40 pmol) or miR-NC and luciferase reporter vectors (2 μg). The luciferase reporter assay was performed with a Dual-luciferase Reporter Assay System (Promega, China).

### Statistical Analysis

Data are shown as mean ± SD. In this study, we used independent-samples *t*-test, χ^2^-test, or one-way ANOVA to analyze the relationship between miRNA expression and clinicopathological factors. All tests were two-tailed, and false discovery rate (FDR) was controlled for multiple comparisons. *p* < 0.05 were considered statistically significant. We applied receiver operating characteristic (ROC) curve analysis to evaluate predictive accuracy of miR-193a-5p for CRC, which was quantified by the area under the ROC curve (AUC). Packages plyr and reshape2 were used for data sorting and restructuring. Ggplot2 was used for visualization of results. Also, GraphPad Prism 8 was applied for statistical analysis.

## Results

### Characterization of Isolated Plasma Extracellular Vesicles From Healthy Individuals and Patients

**Figure 1:**
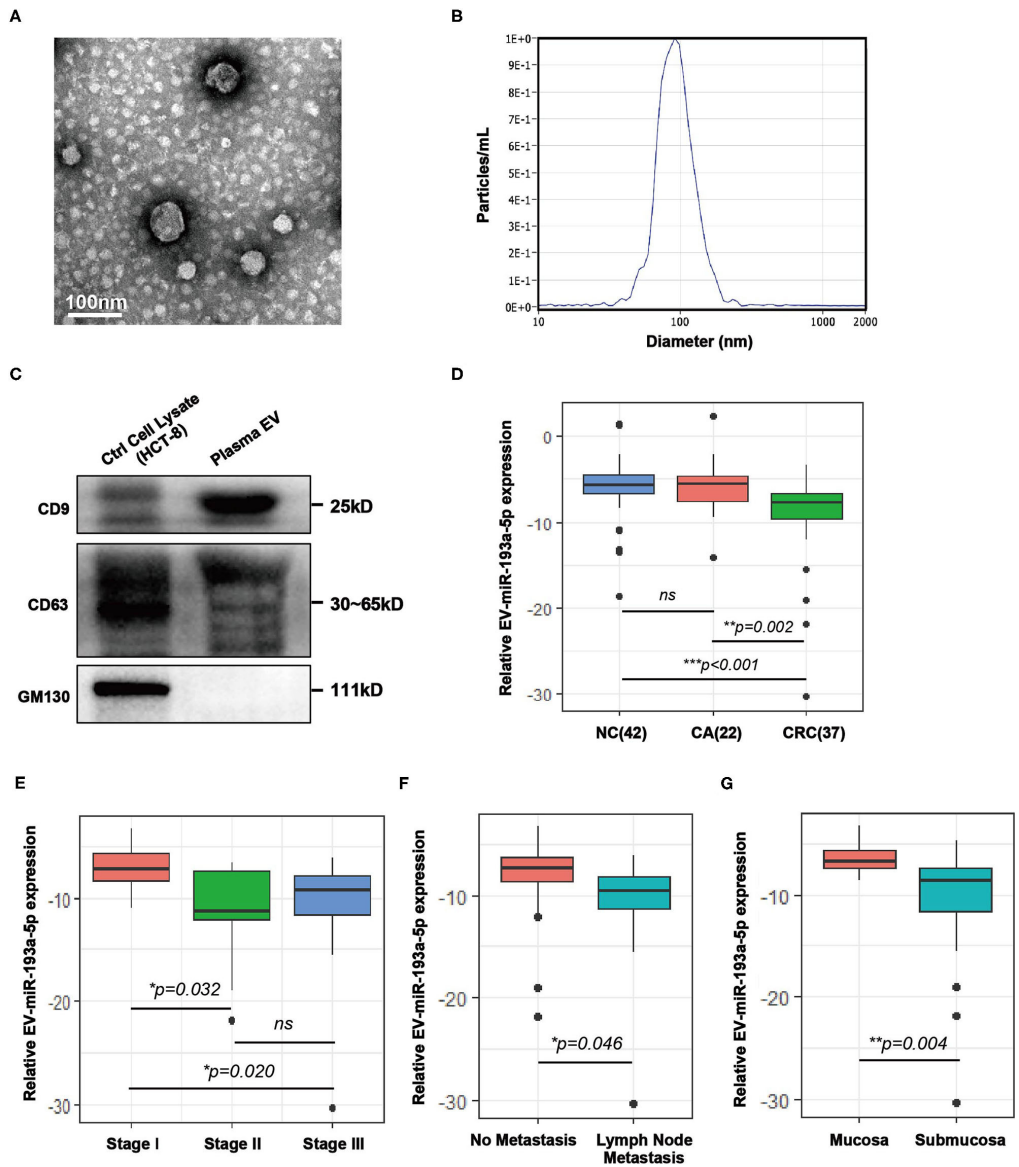
Characterization of extracellular vesicles (EVs) isolated from colorectal cancer (CRC) plasma and comparison of the expression level of plasma EV-miR-193a-5p in CRC, colorectal adenoma (CA) and non-cancerous control (NC). **(A)** Transmission electron microscopy (TEM) revealed the external features of the EVs isolated from plasma. **(B)** Nanoparticle tracking analysis (NTA) suggested that the EVs isolated from plasma were 75–100 nm in diameter. **(C)** Verification of biomarkers of extracellular vesicles by western blotting. Two positive markers (CD9 and CD63) were detected, whereas the negative marker (GM130) was absent. **(D)** Relative expression of EV-miR-193a-5p in plasma of CRC, CA, and NC participants using RT-qPCR (**p < 0.01, ***p < 0.001). **(E)** Relative plasma EV-miR-193a-5p expression in CRC patients at different stages using RT-qPCR (*p < 0.05). **(F)** Relative expression of EV-miR-193a-5p in CRC patients with or without lymph node metastasis by RT-qPCR (*p < 0.05). **(G)** Relative expression of EV-miR-193a-5p in CRC patients with or without submucosa infiltration by RT-qPCR (**p < 0.01).

Plasma samples from healthy individuals and patients with CRC were obtained before treatment and isolated by ultracentrifugation according to published method of Thery (31, 32). To confirm the successful isolation of the plasma EVs, we performed TEM, NTA, and western blotting. TEM images showed that EVs from plasma exhibited vesicular membranes, which are couple-like with diameters of 75–100 nm (Figure 1A). The NTA results showed that the diameter of the EVs concentrated at about 100 nm (Figure 1B). The expression of EV-specific markers including CD63 and CD9 was distinctly observed, and one negative biomarker, GM130, was absent (Figure 1C). Generally, both integrity and purity of isolated EVs from plasma samples were ensured for the following analysis.

### Confirmation of Human Plasma Extracellular Vesicle–microRNAs Using a Bioanalyzer

EVs contain a variety of RNAs, including mRNAs, miRNAs, ncRNAs, transfer RNAs (tRNAs), and ribosomal RNAs (rRNAs) (33). It was vital for our study to acquire purified EV-miRNAs from plasma and then analyze accurately miRNA expression from different samples. The concentration and size of RNAs were measured and analyzed by Agilent 2100 Bioanalyzer. The result showed that there were abundant 25-nucleotide (nt) small RNAs, which represent miRNAs, whereas 18s and 28s rRNAs were hardly detectable (Figure S1A). Thus, the isolated EVs contain miRNAs and are suitable for clinical sample analysis.

### Plasma EV-miR-193a-5p Level in NC, Colorectal Adenoma, and Colorectal Cancer

Here, we evaluated the EV-miR-193a-5p level in plasma samples by RT-qPCR from 101 participants, including NC, CA, and CRC. We found that EV-miR-193a-5p is significantly downregulated in CRC patients as compared with CA and NC individuals (Figure 1D, *p* = 0.002 and *p* < 0.001, respectively). Additionally, no statistical significance was exhibited between CA and NC (ns: *p* = 0.777). These results suggested that EV-miR-193a-5p in plasma could distinguish CRC from precancerous CA and NC but could not distinguish precancerous CA from other non-neoplasm controls.

Among all CRC patients, TNM stage II/III exhibited a significant lower level of miR-193a-5p than among those at TNM stage I (Figure 1E, *p* = 0.032 and *p* = 0.020, respectively), whereas similar expression was observed in the TNM stage II and III patients (Figure 1E, ns: *p* = 0.773). Significantly decreased EV-miR-193a-5p was also observed in the patients with lymph node metastasis and with tumor infiltrating to the submucosa (Figures 1F,G, *p* = 0.046 and *p* = 0.004, respectively). However, no statistical significance was exhibited between CRC subgroups stratified by tumor size, tumor location, age, gender, and Yamada subtype (Figures S2A–E). Among all CA patients, no significant difference in EV-miR-193a-5p level was identified between CA-L and CA-H subgroups (Figure S3A). Additionally, there was no significant difference between CA subgroups stratified by tumor size, distinct tumor location, and Yamada subtype (Figures S3B–D). Among all NC patients, no significant difference was found among those with different diagnoses (Figure S3E).

### Diagnostic Performance of Circulating EV-miR-193a-5p on Colorectal Cancer

**Figure 2:**
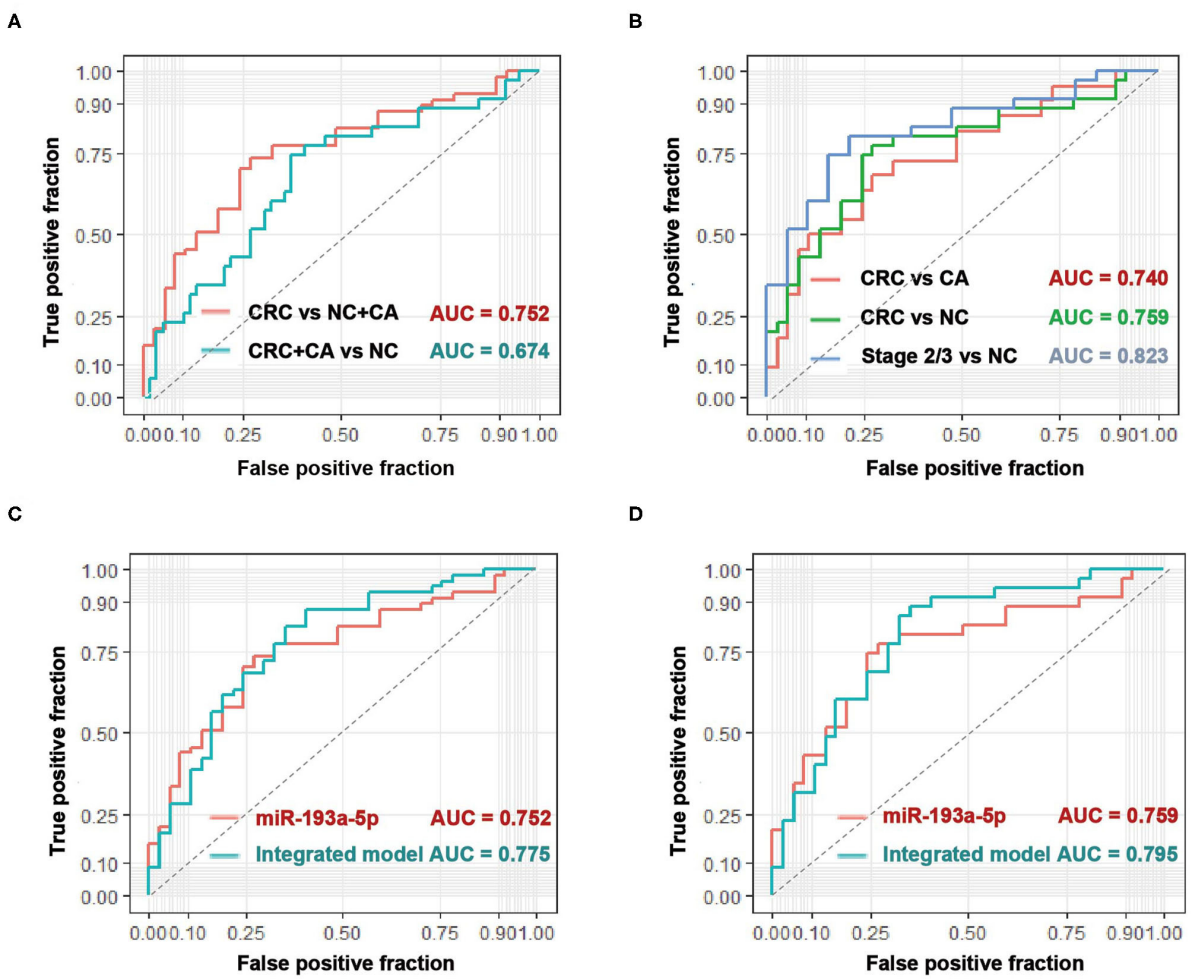
The diagnostic performance of circulating EV-miR-193a-5p as biomarkers in colorectal cancer (CRC). **(A)** Evaluation of EV-miR-193a-5p as a predictive biomarker in CRC vs. colorectal adenoma (CA)+non-cancerous control (NC), and CRC+CA vs. NC. **(B)** Evaluation of EV-miR-193a-5p as a predictive biomarker in CRC vs. CA, CRC vs. NC, and stage II/III CRC vs. NC. **(C)** Comparison between EV-miR-193a-5p and the model-integrated EV-miR-193a-5p and age in predicting CRC from CA+NC. **(D)** Comparison between EV-miR-193a-5p and the model-integrated EV-miR-193a-5p and age in predicting CRC from NC.

With the use of ROC curve analysis, circulating EV-miR-193a-5p had a higher AUC value of 0.752 in distinguishing CRC patients from CA patients and NC participants (CRC vs. CA+NC) and an AUC value of 0.674 in distinguishing CRC and CA patients from NC participants (CRC+CA vs. NC) (Figure 2A), which indicated that miR-193a-5p had a better predictive value in identifying CRC than precancerous lesions. Additionally, the AUC values were 0.740 and 0.759 in distinguishing CRC patients from CA and NC, respectively. In particular, the AUC went up to 0.823 in distinguishing stage II/III CRC from NC participants (Figure 2B).

Age is a well-known and easy-to-access risk factor of CRC (34). Therefore, we tried to integrate age (the cutoff value is 55 years) and circulating EV-miR-193a-5p in a logistic model to achieve a better diagnostic effect in identifying CRC. The AUC value increased from 0.752 to 0.775 in distinguishing CRC from CA and NC (Figure 2C) and increased from 0.759 to 0.795 in distinguishing CRC from NC (Figure 2D).

### miR-193a-5p Is Decreased in Colorectal Cancer Cell Lines and Inhibits Cell Migration Ability

**Figure 3:**
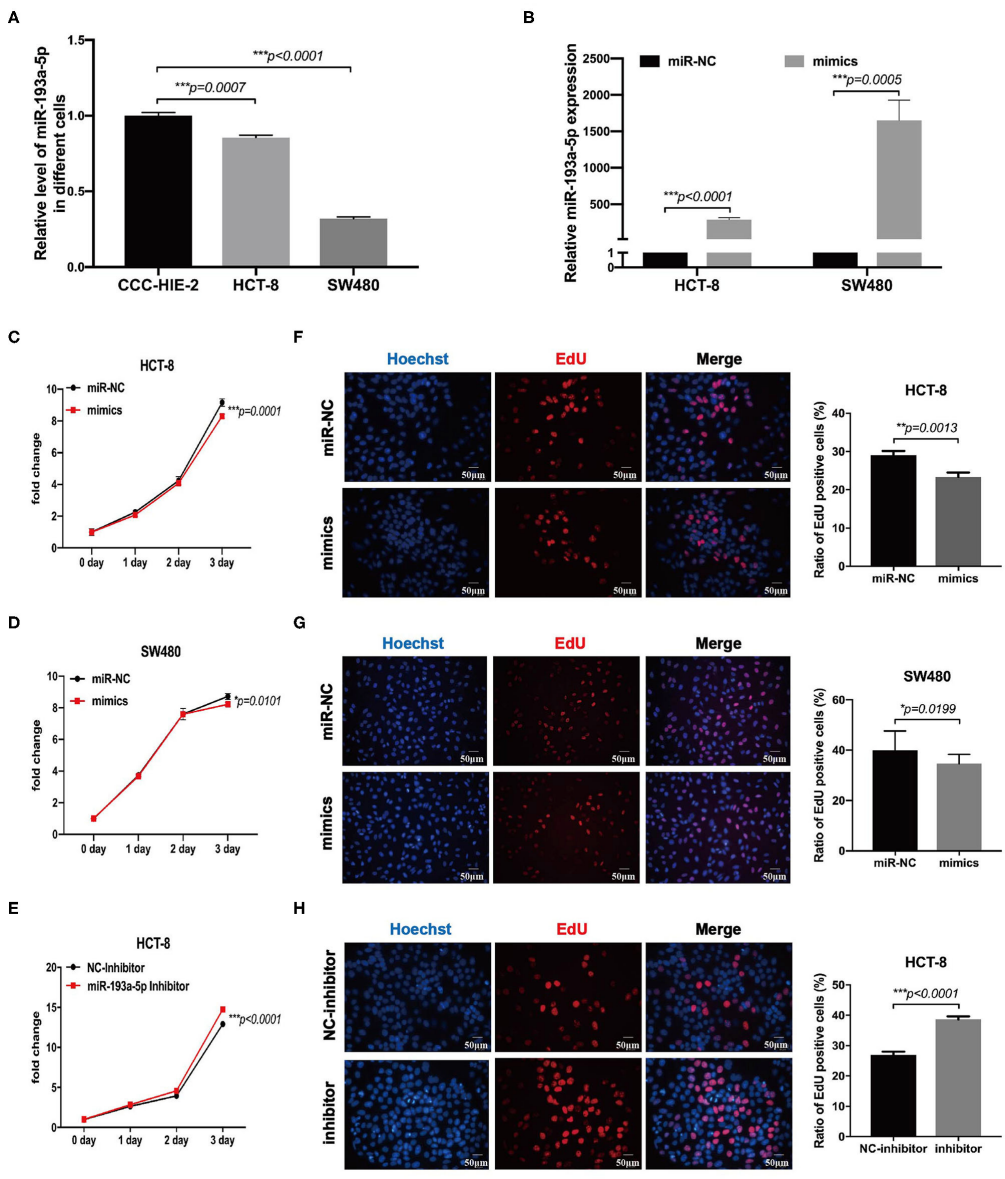
MiR-193a-5p is decreased in colorectal cancer and silently inhibits cell growth and proliferation. **(A)** Comparing the expression level of miR-193a-5p among normal colon epithelial cell and colorectal cancer (CRC) cell lines by RT-qPCR (***p < 0.001). **(B)** The transfection efficiency of HCT-8 and SW480 cell lines by RT-qPCR (***p < 0.001). **(C,D)** Growth curves of HCT-8 and SW480 cells treated with miR-193a-5p mimics and miR-NC (*p < 0.05, ***p < 0.001). **(E)** Growth curves of HCT-8 cells treated with miR-193a-5p inhibitor and inhibitor-NC (***p < 0.001). **(F,G)** EdU staining of HCT-8 and SW480 cells transfected with miR-193a-5p mimics and miR-NC for 48 h (left panel, a group of representative pictures; right panel, average counts from three times of independent test; *p < 0.05, **p < 0.01). **(H)** EdU staining of HCT-8 cells transfected with miR-193a-5p inhibitor and inhibitor-NC for 48 h (left panel, a group of representative pictures; right panel, average counts from three times of independent test; ***p < 0.001).

To choose the appropriate cell lines for function analysis of miR-193a-5p, we firstly compared the relative expression level of miR-193a-5p among different CRC cell lines and one normal colon epithelial cell, CCC-HIE-2. Generally, miR-193a-5p expression was decreased in all CRC cell lines as compared with normal CCC-HIE-2 (Figure 3A, Figure S4A). In a comparison among different CRC cell lines, HCT-116, HT-29, RKO, and SW480 showed lower expression, whereas HCT-8 and HCT-15 showed higher expression of cellular miR-193a-5p. Thus, we chose SW480 and HCT-8 as representative CRC cell lines with high and low miR-193a-5p expression levels, respectively, for further experiments.

Then, miR-NC and miR-193a-5p mimics were transfected into HCT-8 and SW480 cells, and the overexpression efficiency was identified by RT-qPCR (Figure 3B). MTS assay suggested that miR-193a-5p mimic-transduced CRC cell lines showed slightly growth-inhibition effect as compared with cell-transduced miR-NC group (Figures 3C,D). EdU assay reflecting cell mitotic ability suggested that miR-193a-5p overexpression partially inhibited cell proliferation (Figures 3F,G). Subsequently, we transfected HCT-8 cells with miR-193a-5p inhibitor for 48 h. MTS and EdU results showed that cell proliferation ability was promoted (Figures 3E,H).

**Figure 4:**
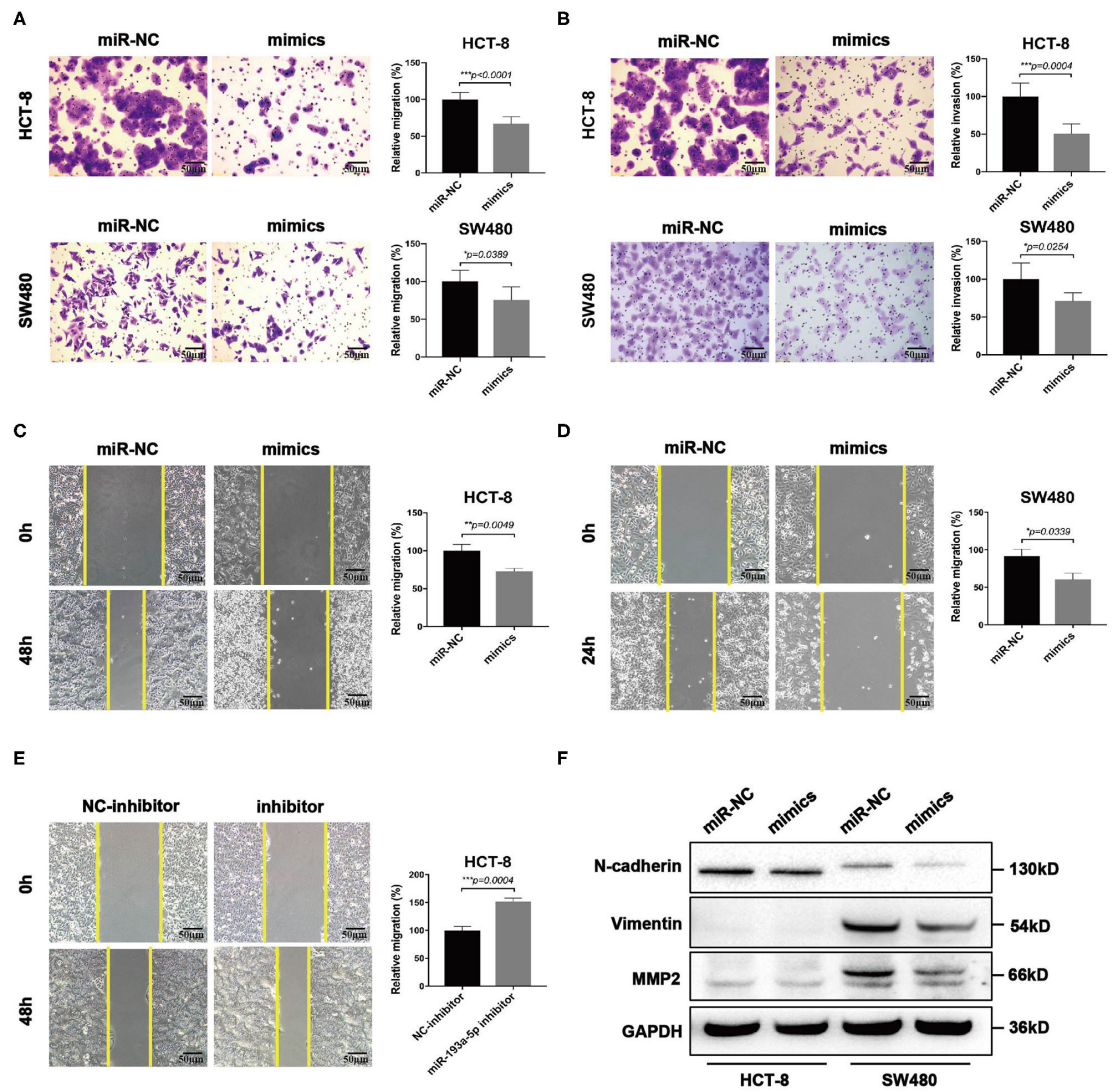
MiR-193a-5p is a tumor-suppressor gene via inhibiting colorectal cancer (CRC) cell migration and invasion. **(A,B)** Cell migration and invasion ability using transwell or Matrigel-coated transwell in HCT-8 and SW480 cells treated with miR-193a-5p mimics (left panel, representative pictures of transwell chambers; right panel, average counts from three times of independent test; *p < 0.05, ***p < 0.001). **(C,D)** Wound healing assay. HCT-8 and SW480 cells were treated with miR-193a-5p mimics for 48 h, and cells were scratched with yellow tips. Images were taken 0, 24, 36, and 48 h after scratching (left panel, representative pictures; right panel, the relative migration distance counts from three times of independent test; *p < 0.05, **p < 0.01). **(E)** Wound healing assay. HCT-8 cells were transfected with miR-193a-5p inhibitor for 48 h and then scratched with yellow tips. Images were taken 0, 24, 36, and 48 h after scratching (left panel, representative pictures; right panel, the relative migration distance counts from three times of independent test; *p < 0.05). **(F)** Epithelial–mesenchymal translation (EMT)-associated genes expression by western blotting, including N-cadherin, Vimentin, and MMP2.

Then we focused on exploring the influence of miR-193a-5p on tumor cell mobility. Transwell assay suggested miR-193a-5p overexpression saliently inhibited cells migration and invasion than miR-NC group (Figures 4A,B). Wound healing assay similarly identified the inhibition on cell migration of miR-193a-5p (Figures 4C,D). Then, we treated HCT-8 cells with miR-193a-5p inhibitor for 48 h; wound healing assay suggested that after treatment with non-serum medium for 48 h, the mobility of HCT-8 cell was increased evidently (Figure 4E). Among the phenotype results above elicited, miR-193a-5p potentially functioned as a tumor suppressor in CRC cell lines and is mainly involved in repressing CRC migration and invasion. Our results also identified that high expression of miR-193a-5p after transfection decreased the protein levels of some EMT-related genes, including N-cadherin, Vimentin, and MMP2 (Figure 4F).

### CUX1 and ITSN1 Are the Target Genes of miR-193a-5p and Associated With Poor Prognosis of Colorectal Cancer

**Figure 5:**
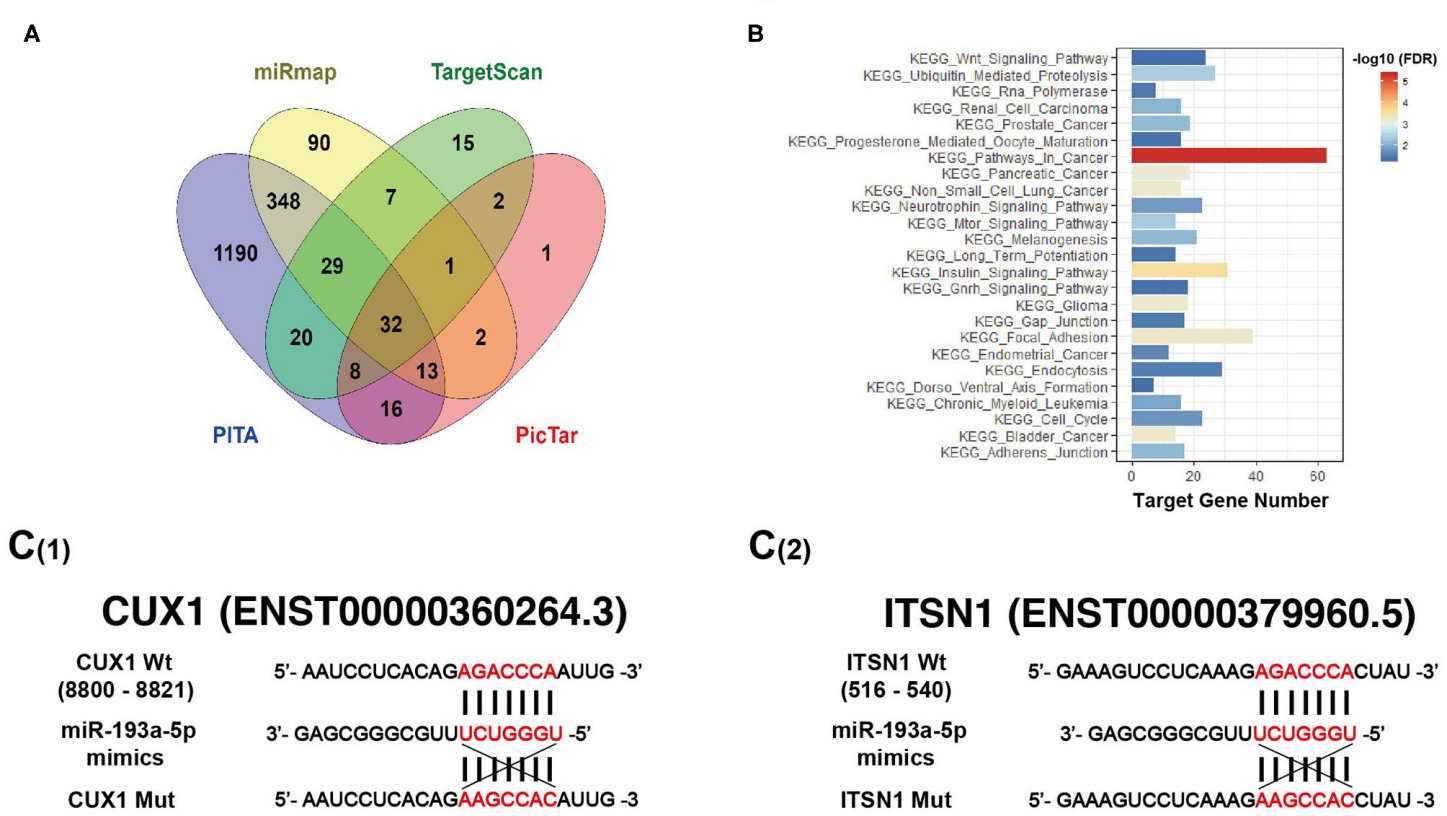
Bioinformatic analysis predicts target genes of miR-193a-5p. **(A)** Four miRNAs targeting prediction websites were used to select target genes of miR-193a-5p. **(B)** Gene set enrichment analysis (GSEA) showed miR-193a-5p targeting genes' co-associated signaling pathways and Kyoto Encyclopedia of Genes and Genomes (KEGG) pathways in cancer is the most genes related. **(C)** Schematic representation of the 3′-UTR of CUX1 and ITSN1 with the predicted target sites for miR-193a-5p.

It is well-known that miRNAs affect the expression of their target genes to exert biological functions on tumor behavior. Bioinformatic analysis was performed using four miRNAs targeted predicting tools (miRmap, TargetScan, PITA, and PicTar), and the Venn diagram showed that 32 genes were co-predicted by all the above four databases (Figure 5A). Furthermore, enriched Kyoto Encyclopedia of Genes and Genomes (KEGG) pathways exhibited target genes of miR-193a-5p significantly focused on cancer-associated KEGG pathways (Figure 5B). Among all 32 putative target genes of miR-193a-5p, we identified four tumor-associated genes, including CUX1, ITSN1, OLA1, and RAP2A. By RT-qPCR and western blotting experiments, we found that only the expression of CUX1 and ITSN1 was downregulated under overexpression of miR-193a-5p (Figures S4B,C). Thus, we finally chose CUX1 and ITSN1 for miR-193a-5p target genes in the subsequent analysis.

**Figure 6:**
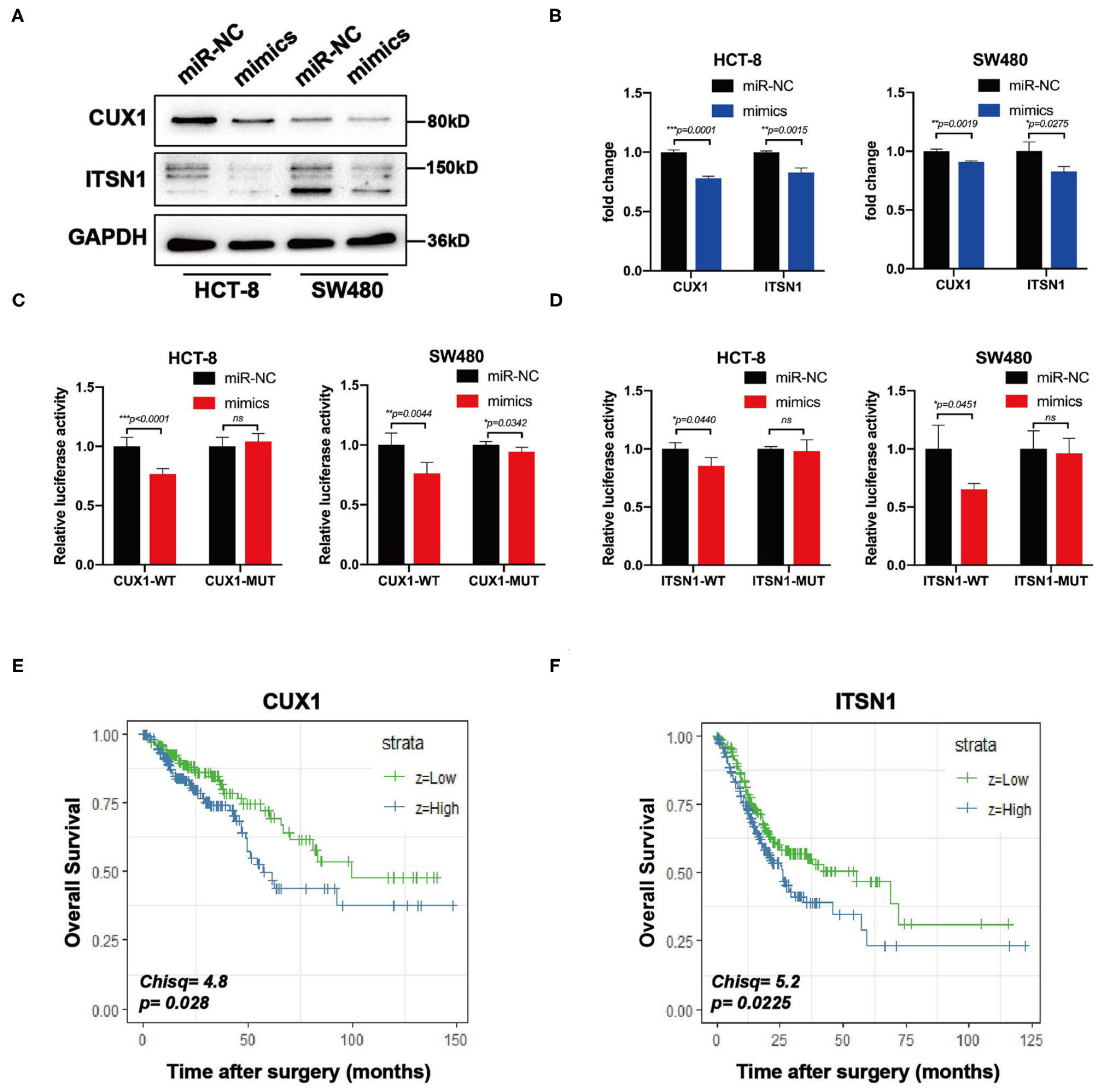
CUX1 and ITSN1 are target genes of miR-193a-5p and associated with poor prognosis of colorectal cancer (CRC). **(A)** The protein expression analysis of CUX1 and ITSN1 in CRC cells after transfection with miR-193a-5p mimics or miR-NC. **(B)** The mRNA expression analysis of CUX1 and ITSN1 in HCT-8 and SW480 cells after transfection with miR-193a-5p mimics or miR-NC (*p < 0.05, **p < 0.01, ***p < 0.001). **(C,D)** Dual-luciferase reporter assay. Reporter constructs containing either wild-type CUX1/ITSN1 3′-UTR or CUX1/ITSN1 3′-UTR with mutation at the predicted miR-193a-5p target sequence were transfected into CRC cells, along with miR-193a-5p mimics or miR-NC; the relative luciferase activity was assayed (*p < 0.05, **p < 0.01, ***p < 0.001). **(E,F)** The overall survival (OS) of CRC patients stratified by CUX1 and ITSN1 expression levels.

Bioinformatic analysis showed that the 3′-UTRs of CUX1 and ITSN1 mRNAs contain a highly conserved binding site for miR-193a-5p seed sequence (Figure 5C). To further confirm whether CUX1 and ITSN1 are the target genes, we performed western blotting and RT-qPCR. The protein level of CUX1 and ITSN1 was significantly decreased by miR-193a-5p overexpression in CRC cell lines (Figure 6A). However, the mRNA level of two genes was slightly decreased (Figure 6B). Next, to further validate that these two genes are direct targeting genes of miR-193a-5p, we constructed luciferase reporter vector containing wild-type (WT) CUX1 and ITSN1 3′-UTRs with miR-193a-5p binding site (WT) and containing the mutant 3′-UTRs (MUT). Results showed that miR-193a-5p decreased the luciferase activity of CUX1 and ITSN1-3′-UTRs WT reporter but did not affect the MUT reporter in two CRC cell lines (Figures 6C,D). In summary, these data suggested that CUX1 and ITSN1 are direct targeting genes of miR-193a-5p in CRC. Additionally, analysis of data from The Cancer Genome Atlas (TCGA) suggested that higher CUX1 and ITSN1 were correlated with a worse overall survival in CRC (CUX1, χ^2^ = 4.8, *p* = 0.028; ITSN1, χ^2^ = 5.2, *p* = 0.0225; Figures 6E,F).

## Discussion

Liquid biopsy is a promising non-invasive method for detecting and analyzing cancer-specific biomarkers. The nucleic acids and proteins in the circulating EVs are more stable and representative, which were very suitable for liquid biopsy. Previous studies found that EVs provide a more consistent source of miRNAs and reflect their cell of origin (35). Here, we found that decreased EV-miR-193a-5p in plasma could be helpful for the diagnosis of CRC patients, particularly in TNM stage II/III. Additionally, we observed no significance of circulating EV-miR-193a-5p between CA patients and NC participants, so the plasma EV-miR-193a-5p decreasing could occur during the CA cancerization. Age is another important risk factor for cancer. Here, we found that integrating age with plasma EV-miR-193a-5p could achieve a better AUC of 0.795 in identifying CRC patients from NC. This integrated model could serve as an economical tool with considerable sensitivity and specificity in clinical practice as compared with previously published miRNA panels in CRC diagnosis because only one biological indicator was examined.

The lower EV-miR-193a-5p level in plasma could also indicate lymph node metastasis in CRC patients. In CRC patients with lymph node metastasis, the plasma EV-miR-193a-5p level was significantly lower. Interestingly, Zhang et al. demonstrated that tissue miR-193a-5p was lower in CRC patients with lymph node metastasis (24). Qu et al. revealed that a decreased serum miR-193a-5p could help predict the probability of lymph node metastasis and the risk of CRC progression (27). Thus, the reduced miR-193a-5p expression in CRC patients is consistent in plasma-derived EVs, tissue, and serum. As a tumor-suppressive gene, the cellular origin of EV-miR-193a-5p and circulating miR-193a-5p is not clear. The downregulation of the release and expression of miR-193a-5p in normal tissues by some cancer cell-derived signaling molecules could be a possible explanation. In contrast, Teng et al. found that cancer cells released EVs with higher expression of tumor-suppressor gene, such as miR-193a into circulating (36). In more advanced colon cancer patients, especially with liver metastasis, higher levels of tumor-suppressor miRNAs encapsulated in the EVs were found in the peripheral blood. It is well-known that miRNAs were selectively encapsulated into EVs via specific molecular pathways, and the various sorting mechanisms of EV-miRNAs may explain the inconsistency of EVs-miRNA expression among different miRNA species.

We further explored the role of miR-193a-5p in CRC. We found that miR-193a-5p suppressed tumor migration and invasion via targeting EMT associated genes *in vitro*. Four bioinformatic prediction tools helped us to select CUX1 and ITSN1, as functioned target genes for miR-193a-5p. The role of CUX1 in cancer is complex and still controversial. Elevated expression of CUX1 was observed and positively associated with poor prognosis in CRC, high-grade breast cancer, and pancreatic cancer (37). CUX1 can regulate motility-associated gene expression to stimulate cancer migration and invasion, such as Wnt/β-catenin, snail, and slug (38, 39). In the cancers of the large intestine, copy number variation analysis evidenced that frequent copy number gain of CUX1 was observed and associated with tumor aggressiveness (40). Another target gene, ITSN1, is highly conserved gene and regulates endocytosis and multiple signaling pathways. It was reported to be involved in human tumorigenesis, including neuroblastomas, glioblastomas, and lung cancer (41). Recent work showed ITSN1 stimulated glioma cell migration and invasion via regulating cofilin, LIMK, PAK, FAK, integrin β1, and MMP9 (42). Yet there were no reports on exploring the expression level and function in CRC cancer. Here, we identified that CUX1 and ITSN1 might contribute to the inhibition on cell motility by miR-193a-5p. We also showed that the higher CUX1 and ITSN1 expression was inversely correlated with overall survival in CRC. Thus, we confirmed that miR-193a-5p played a critical role in downregulating oncogene expression and predicted a good prognosis of CRC patients.

There are also some limitations in our study: (1) the impact of circulating EV-miR-193a-5p on the metastasis of CRC in *vitro* or *vivo* should be further evaluated, when a plasma EV isolation method with much higher recovery was available. (2) Direct comparison between the performance of circulating miR-193a-5p and that of EV-miR-193a-5p in CRC diagnosis would be also very helpful to demonstrate the necessity of EV isolation procedure in miR-193a-5p quantification, even though one of our previous studies (32) and increasing evidence proved that EV-miRNA was more informative than serum/plasma miRNAs in many cancers (35, 43). (3) A larger cohort with more advanced CRC patients is also needed to reveal the prognostic role of circulating EV-miR-193a-5p.

In conclusion, we revealed a lower expression of plasma EV-miR-193a-5p in CRC patients as compared with precancerous CA and NC individuals. The EV-miR-193a-5p level is especially lower in CRC patients with lymph node metastasis and tumor infiltration of the submucosa. Additionally, we developed an integrated diagnostic model combining EV-miR-193a-5p level and age, which could be an efficient tool in detecting CRC high-risk individuals.

## Data Availability Statement

All datasets generated for this study are included in the article/Supplementary Material.

## Ethics Statement

The studies involving human participants were reviewed and approved by the ethics committee of Beijing Friendship Hospital. The patients/participants provided their written informed consent to participate in this study.

## Author Contributions

LM and SZha conceived and designed the study. RW and LC performed all experiments. DQ and QG helped to collect, reformat, and analyze the primary data. RW and LM drafted the manuscript. SZhu, PL, LM, and SZha helped to revise the manuscript. All authors read and approved the final manuscript.

## Conflict of Interest

The authors declare that the research was conducted in the absence of any commercial or financial relationships that could be construed as a potential conflict of interest.
