# Supplementary material for: Liquid Biopsy of Extracellular Vesicle-Derived miR-193a-5p in Colorectal Cancer and Discovery of Its Tumor-Suppressor Functions
Source: Front Oncol. 2020 Aug 18;10:1372. doi: 10.3389/fonc.2020.01372 (PMC7461920; doi:10.3389/fonc.2020.01372)
Supplement: Supplementary file 1 [file Data_Sheet_1.PDF]

S.A

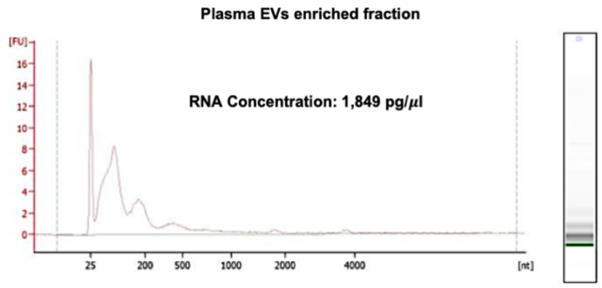

Figure S1. Confirmation of human plasma EV-miRNAs.

(A) Representative result of plasma EV enriched fractions derived RNAs by Agilent 2100 Bioanalyzer (25nt means miRNAs).

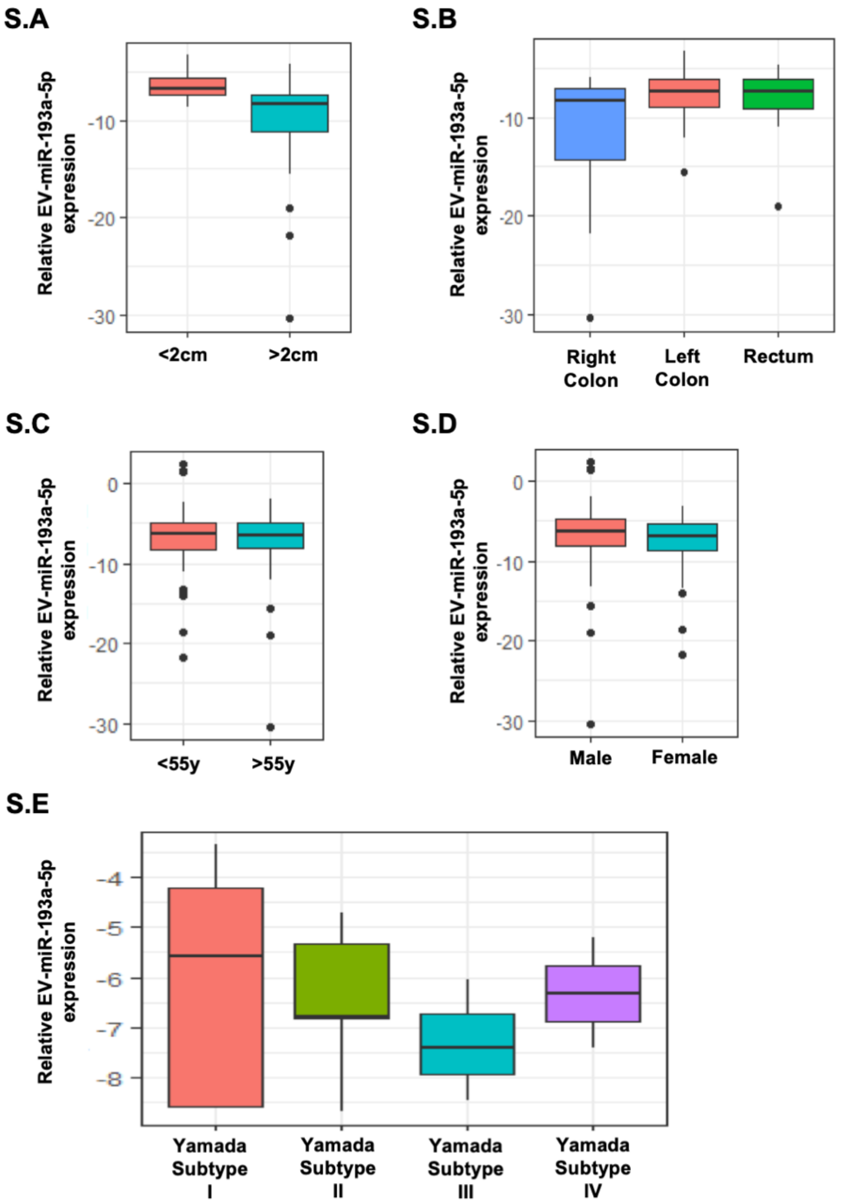

Figure S2. Comparing the plasma EV-miR-193a-5p expression in CRC patients at different subgroups.

(A) Tumor size (left: <2cm; right: >2cm). **(B)** Tumor location (left: Right colon; middle: Left colon; right: Rectum). **(C)** Patients age (left: <55 years; right:>55 years). **(D)** Patients gender (left: Male; right: Female). **(E)** Yamada Subtype (I, II, III and IV).

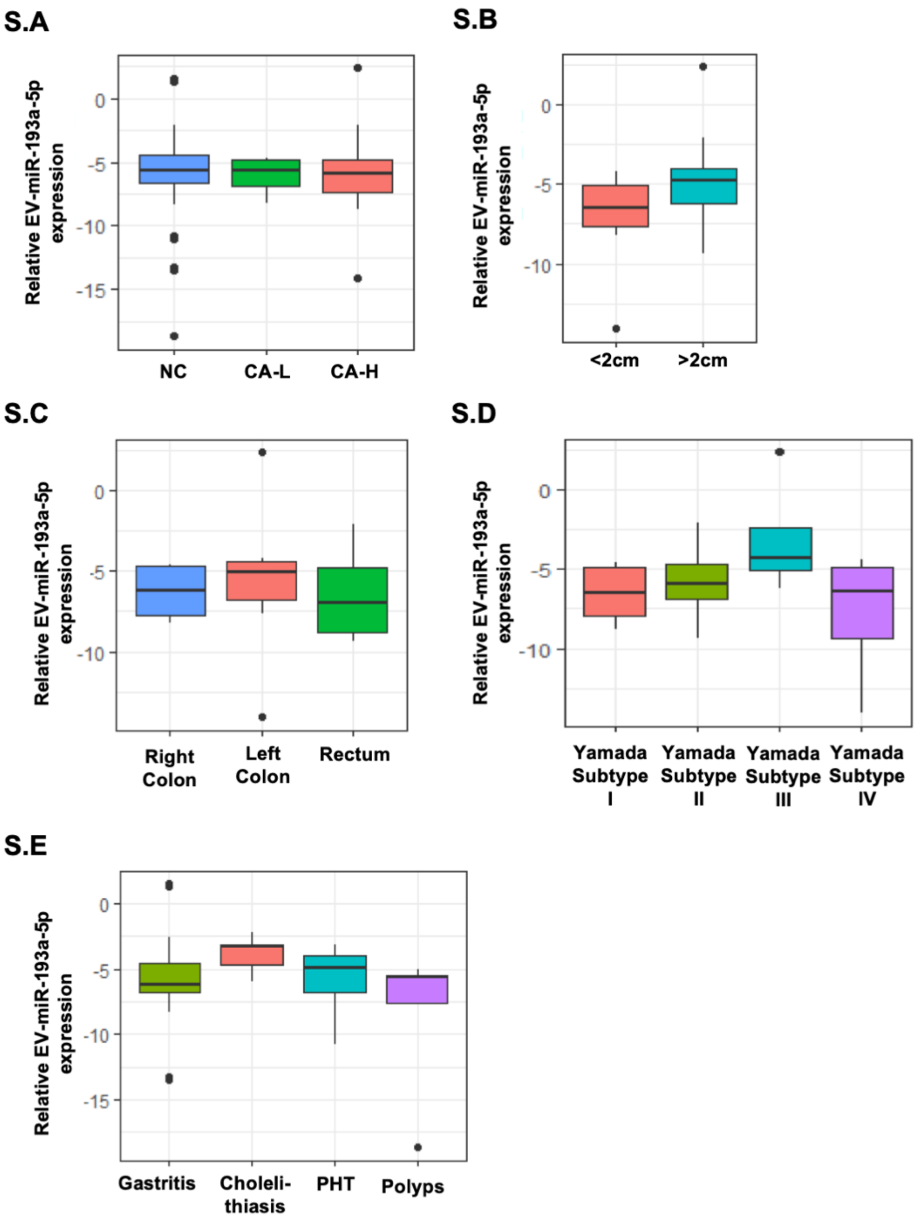

Figure S3. Comparing the plasma EV-miR-193a-5p expression in NC and CA patients at different subgroups.

(A) NC and CA subgroups (CA-Low grade, CA-High grade). **(B)** Tumor size (left: <2cm; right: >2cm). **(C)** Tumor location (left: Right colon; middle: Left colon; right: Rectum). **(D)** Yamada Subtype (I, II, III and IV). **(E)** Other digestive diseases (Gastritis, Cholelithiasis, PHT (portal hypertension), Polyps).

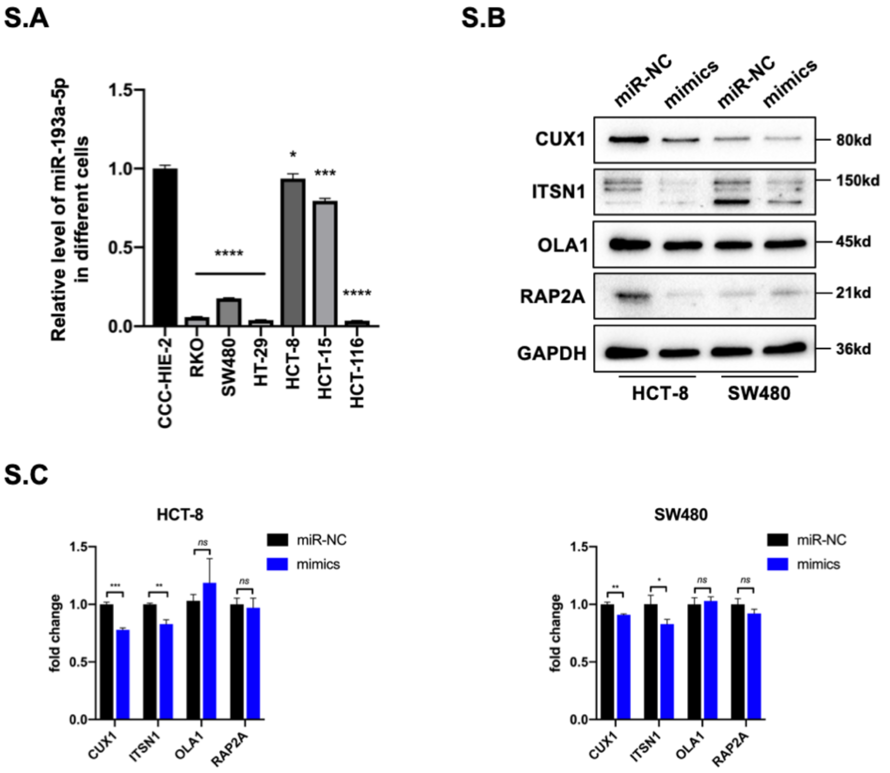

Figure S4. miR-193a-5p is decreased in different CRC cell lines, and CUX1 and ITSN1 are its' target genes.

(A) Comparing the relative expression level of miR-193a-5p among normal colon epithelial cell (CCC-HIE-2) and different CRC cell lines by RT-qPCR (**p<0.05, ***p<0.001, ****p<0.0001*). **(B)** The protein expression analysis of 4 tumor associated target genes, including CUX1, ITSN1, OLA1, and RAP2A, in CRC cells after transfection with miR-193a-5p mimics or miR-NC. **(C)** The mRNA expression analysis of 4 tumor associated genes, including CUX1, ITSN1, OLA1, and RAP2A, in HCT-8 and SW480 cells after transfection with miR-193a-5p mimics or miR-NC (**p<0.05, **p<0.01, ***p<0.001*).
