# Supplementary figures and images for: Liquid Biopsy of Extracellular Vesicle-Derived miR-193a-5p in Colorectal Cancer and Discovery of Its Tumor-Suppressor Functions
Source: Front Oncol. 2020 Aug 18;10:1372. doi: 10.3389/fonc.2020.01372 (PMC7461920; doi:10.3389/fonc.2020.01372)

Extended Data Figure 1C

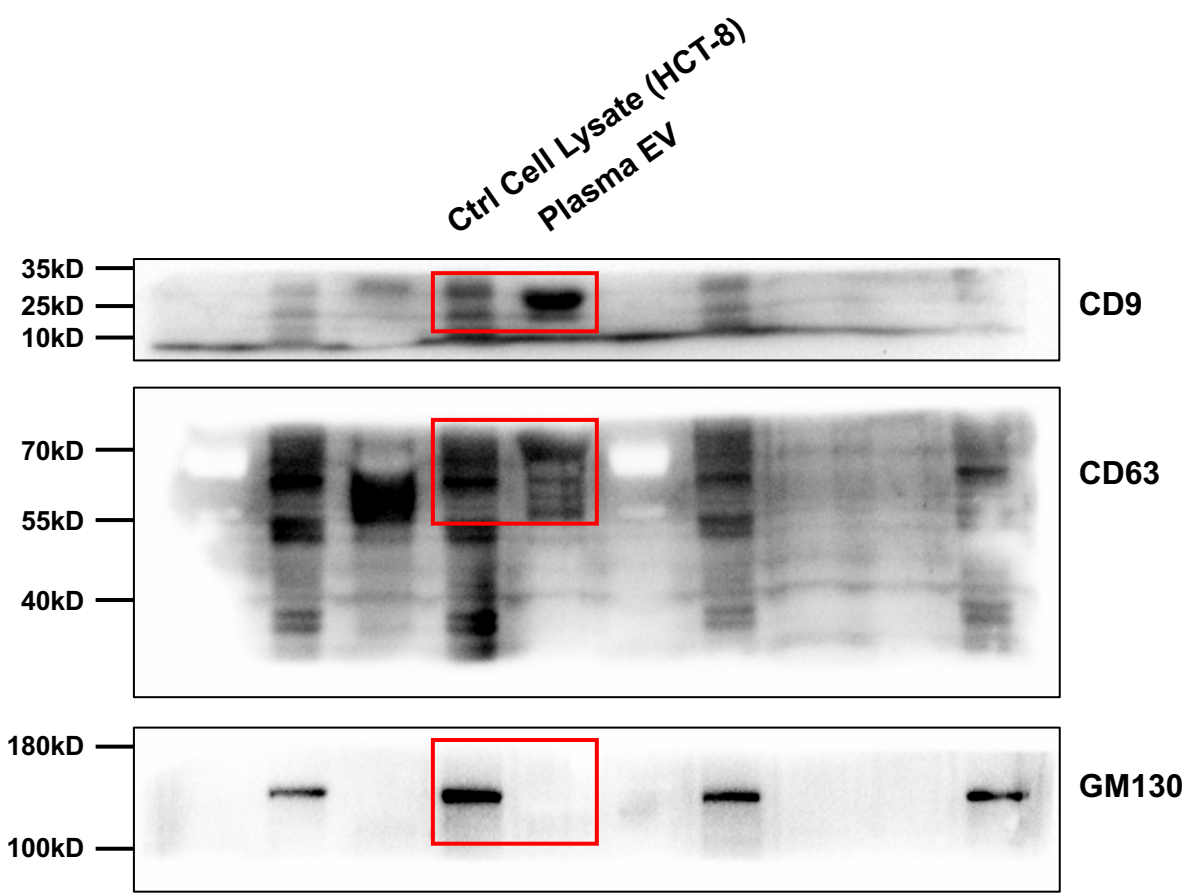

Extended Data Figure 4F

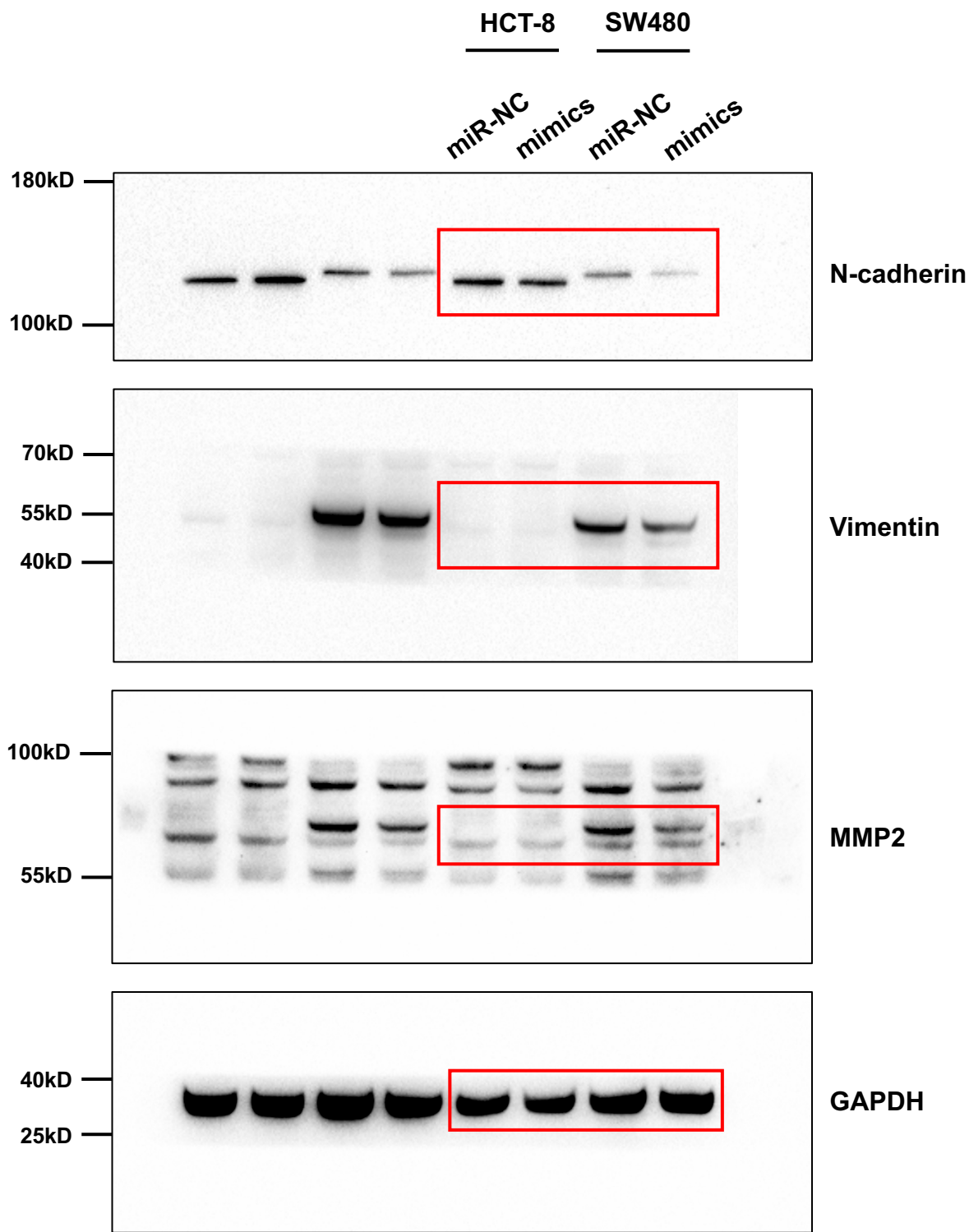

Extended Data Figure 6A

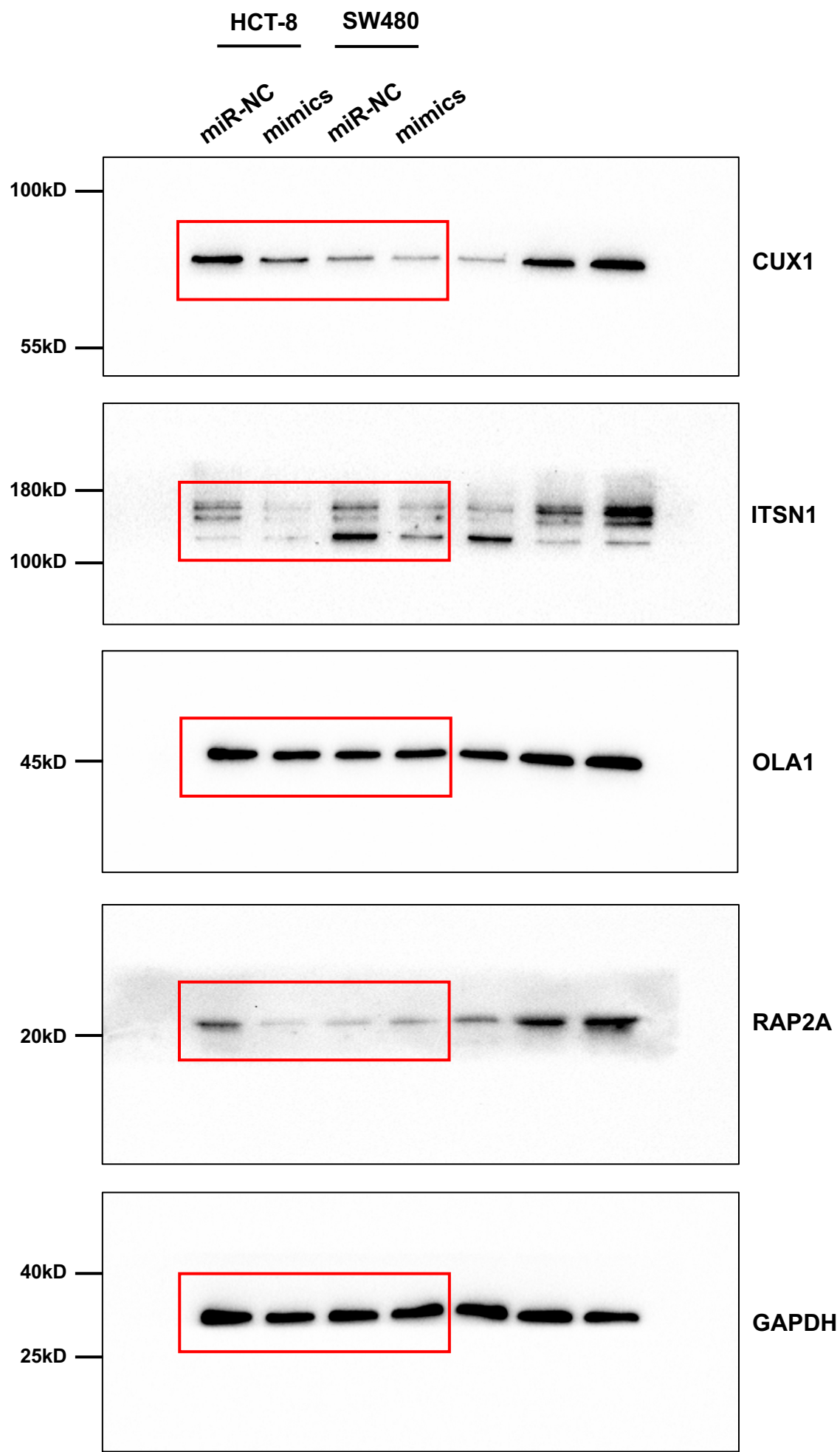

Supplement: Supplementary file 2 [file Data_Sheet_2.PDF]
